# Prevalence of hypovitaminosis D and predictors of vitamin D status in Italian healthy adolescents

**DOI:** 10.1186/1824-7288-40-54

**Published:** 2014-06-05

**Authors:** Francesco Vierucci, Marta Del Pistoia, Margherita Fanos, Paola Erba, Giuseppe Saggese

**Affiliations:** 1 Pediatric Unit, Campo di Marte Hospital, Via Ospedale 1, 55100 Lucca, Italy; 2 Pediatric Clinic, University-Hospital Pisa, Pisa, Italy; 3 Nuclear Medicine Unit, University-Hospital Pisa, Pisa, Italy

**Keywords:** Adolescents, Hypovitaminosis D, Vitamin D deficiency, Vitamin D insufficiency, Parathyroid hormone

## Abstract

**Background:**

Vitamin D plays an important role in health promotion during adolescence. Vitamin D deficiency and insufficiency are common in adolescents worldwide. Few data on vitamin D status and risk factors for hypovitaminosis D in Italian adolescents are currently available.

**Methods:**

25-hydroxyvitamin D (25-OH-D) and parathyroid hormone (PTH) levels were evaluated in 427 Italian healthy adolescents (10.0-21.0 years). We used the following cut-off of 25-OH-D to define vitamin D status: deficiency < 50 nmol/L; insufficiency 50-75 nmol/L; sufficiency ≥ 75 nmol/L. Hypovitaminosis D was defined as 25-OH-D levels < 75.0 nmol/L and severe vitamin D deficiency as 25-OH-D levels < 25.0 nmol/L. We evaluated gender, residence, season of blood withdrawal, ethnicity, weight status, sun exposure, use of sunscreens, outdoor physical activity, and history of fractures as predictors of vitamin D status.

**Results:**

Enrolled adolescents had a median serum 25-OH-D level of 50.0 nmol/L, range 8.1-174.7, with 82.2% having hypovitaminosis D. Vitamin D deficiency and insufficiency were detected in 49.9% and 32.3% of adolescents, respectively. Among those with deficiency, 38 subjects were severely deficient (38/427, 8.9% of the entire sample). Non-white adolescents had a higher prevalence of severe vitamin D deficiency than white subjects (6/17-35.3% vs 32/410-7.8% respectively, p = 0.002). Logistic regression showed increased risk of hypovitaminosis D as follows: blood withdrawal taken in winter-spring (Odds ratio (OR) 5.64) compared to summer-fall period; overweight-obese adolescents (OR 3.89) compared to subjects with normal body mass index (BMI); low sun exposure (OR 5.94) compared to moderate-good exposure and regular use of sunscreens (OR 5.89) compared to non regular use. Adolescents who performed < 3 hours/week of outdoor exercise had higher prevalence of hypovitaminosis D. Gender, residence, and history of fractures were not associated with vitamin D status. Serum 25-OH-D levels were inversely related to PTH (r = -0.387, p < 0.0001) and BMI-SDS (r = -0.141, p = 0.007). 44/427 (10.3%) adolescents showed secondary hyperparathyroidism.

**Conclusions:**

Italian adolescents have high prevalence of vitamin D deficiency and insufficiency. Pediatricians should tackle predictors of vitamin D status, favoring a healthier lifestyle and promoting supplementation in the groups at higher risk of hypovitaminosis D.

## Introduction

An optimal vitamin D status is considered important for health promotion during pediatric age and later in life. Despite this well-known assumption, vitamin D deficiency and insufficiency are still reported as a frequent problem in children and adolescents worldwide [1,2]. In Italy, there are limited data on the prevalence of vitamin D deficiency among healthy Italian adolescents. Furthermore, there is paucity of data on the predictors of vitamin D status in Italian pediatric population. Factors known to influence vitamin D status include sunshine exposure, skin pigmentation, seasonality, body mass index (BMI) and dietary factors, particularly vitamin D supplementation and intake of vitamin D rich food [3,4]. Adolescents are particularly at risk of hypovitaminosis D due to the increasing tendency to sedentary lifestyle, with excessive computer and TV use. Being sedentary reduces time spent outdoor in sunlight on one side and on the other side it increases the risk of obesity, which is another risk factor for hypovitaminosis D [4-6]. Adolescence is a period of rapid growth and bone mineral accrual [7,8] during which vitamin D status should be maintained within the optimal range. Indeed, vitamin D plays a role in the achievement of peak bone mass (PBM) during adolescence [7,9,10]. The aims of the present study were therefore to assess vitamin D status in Italian adolescents living in the province of Pisa, Central Italy (latitude 43°43′N) and to identify the risk factors for hypovitaminosis D.

## Methods

We enrolled 427 Italian adolescents (10.0-21.0 years) recruited from the Pediatric Clinic of the University of Pisa, living in the Northwestern area of Tuscany, Central Italy (latitude 43°43′N) during a period of 24 months (October 2010-September 2012). None of the adolescents was affected by any disease of phospho-calcic metabolism and none had received supplementation with vitamin D in the previous 12 months.

Measurements of standing height and body weight of subjects were assessed using a wall-mounted stadiometer and a mechanical balance. Trained personnel conducted the measurements in a standardized manner and obtained both height and weight as the mean of three measurements. BMI was calculated as weight (Kg) divided by height squared (m^2^). Height, weight and BMI were expressed as Z-score according to the LMS method of Cole et al. [11]. Weight status was categorized in normal, overweight and obese according to Cole et al. for subjects under 18 years [12] and according to the World Health Organization for subjects aged 18-21 years (BMI < 25.0, 25.0-29.9 and ≥ 30.0 Kg/m^2^, respectively). Auxological evaluation was performed in 366/427 (85.7%) adolescents (Table 1).

**Table 1:** Characteristics of the subjects

	**n**	**Median (range)**
Age, years	427	14.3 (10.0; 21.0)
Height, SDS	366	-0.4 (-2.1; 2.4)
Weight, SDS	366	0.4 (-2.1; 3.8)
BMI, SDS	366	0.8 (-2.2; 3.9)
Calcium intake, mg/day	277	609.6 (200.2; 1,280.9)
Vitamin D intake, IU/day	277	14.8 (2.4; 282.8)

Dedicated personnel carried out a detailed interview on background information about lifestyle, particularly on sun exposure, place of residence and outdoor physical exercise and general information about dietary habits. 277/427 (64.9%) adolescents consented to the interview. Sun exposure was evaluated in terms of days of significant exposure to sunlight during the period of the study (if subjects enrolled during summer) or during the summer previous to the enrolment (if subjects enrolled during fall, winter and spring). At our latitude (43°44′N), cutaneous synthesis of vitamin D takes place only in the summer months (May-September) [13]. We defined significant exposure to sunlight as the exposure of the arms and legs for 15 minutes between 10 a.m. and 3 p.m. without the application of sunscreens [14], identifying the following three categories: poor (<15 days), moderate (15-30 days) or good (≥30 days) sun exposure. The use of sunscreens was also investigated and defined as regular or irregular, defining regular the use of a sunscreen with a sun protection factor (SPF) ≥ 15 applied at least 30 minutes before sun exposure and subsequently every 2 hours [15]. The place of residence was categorized as urban or rural. Dietary habits and daily dietary intakes of vitamin D and calcium were assessed using the software WinFood© 1.5 (Medimatica, Teramo, Italy). Food models or serving containers were used to assist in estimating serving size.

25-hydroxyvitamin D (25-OH-D), 1,25-dihydroxyvitamin D (1,25(OH)_2_D), and parathyroid hormone (PTH) levels were evaluated on a fasting blood sample obtained by blood withdrawal. 25-OH-D and PTH levels were evaluated in all the subjects while 1,25(OH)_2_D levels were available in 304/427 (71.2%) adolescents. We used the following cut-off of 25-OH-D to define vitamin D status: vitamin D deficiency < 50 nmol/L (20 ng/mL); vitamin D insufficiency 50-75 nmol/L (20-30 ng/mL); vitamin D sufficiency ≥ 75 nmol/L (30 ng/mL), according to the Endocrine Society [16] and the Society for Adolescent Health and Medicine [8]. Thus hypovitaminosis D was defined in presence of 25-OH-D levels < 75.0 nmol/L (30 ng/mL). Furthermore, severe vitamin D deficiency was defined as 25-OH-D levels < 25.0 nmol/L (10 ng/mL) [17]. PTH levels ≥ 65.0 ng/L were suggestive of hyperparathyroidism.

25-OH-D and 1,25(OH)_2_D levels were measured by radioimmunoassay (25-Hydroxyvitamin D ^125^I RIA Kit, DiaSorin, intra- and inter-assay CV = 11%; 1,25-Dihydroxyvitamin D ^125^I RIA Kit, DiaSorin, intra-assay CV = 8.6%, inter-assay CV = 11.9%). Biologically active intact hPTH 1-84 was measured by immunoradiometric assay (N-tact® PTH SP Irma Kit, DiaSorin, intra-assay CV = 3.6%, inter-assay CV = 4.9%). Written informed consent to the study was obtained from all enrolled subjects or their parents, as requested. The study was approved by the Ethics Committee for Human Investigation at our University.

Non parametric Mann-Whitney test and Kruskal-Wallis test were used to compare groups (variables not normally distributed). Data were reported as median and range. Chi-squared test was used to compare groups of categorical variables. A multiple logistic regression analysis was performed to study the association between each presumed risk factor and vitamin D status. Spearman’s correlation coefficients were adopted to explore the relationship between serum 25-OH-D levels and age, BMI, biochemical findings and dietary calcium and vitamin D intake. All statistical analyses were carried out using the SPSS (Statistical Package of Social Sciences, Chicago, IL, USA) for Windows software program version 19.0. A p value <0.05 was considered significant.

## Results

Enrolled Italian adolescents showed a median serum 25-OH-D level of 50.0 nmol/L, range 8.1-174.7, with 82.2% (351/427) having hypovitaminosis D. Particularly, vitamin D deficiency and insufficiency were detected in 49.9% (213/427) and 32.3% (138/427) of adolescents, respectively. Among those with deficiency, 38 subjects were severely deficient (38/427, 8.9% of the entire sample).

25-OH-D levels and vitamin D status in relation to the presumed risk factors for hypovitaminosis D are summarized in Table 2. Season of blood withdrawal, ethnicity, weight status, sun exposure, use of sunscreens and outdoor physical exercise were significantly associated with vitamin D status in Italian adolescents while gender, residence and history of fractures were not. Subjects evaluated in winter and spring, overweight or obese adolescents, individuals reporting low sun exposure, regular use of sunscreens or performing outdoor physical exercise < 3 hours/week had median 25-OH-D levels in the range of vitamin D deficiency and a significantly increased prevalence of hypovitaminosis D. Particularly, non-white adolescents had extremely reduced median 25-OH-D levels with only one subject with vitamin D sufficiency.

**Table 2:** Serum 25-OH-D levels and vitamin D status related to presumed risk factors for hypovitaminosis D

	**n/total (%)**	**25-OH-D, nmol/L**	**p**	**n/total (%) with vitamin D deficiency**	**n/total (%) with vitamin D insufficiency**	**n/total (%) with vitamin D sufficiency**	**p**
		**Median (range)**					
*Gender*							
Male	213/427 (50.0)	48.3 (10.0; 174.7)	0.438	111/213 (52.1)	62/213 (29.1)	40/213 (18.8)	0.366
Female	214/427 (50.0)	50.4 (8.1; 174.3)		102/214 (47.7)	76/214 (35.5)	36/214 (16.8)	
*Residence*							
Urban	238/427 (55.7)	48.0 (10.8; 174.7)	0.481	124/238 (52.1)	73/238 (30.7)	41/238 (17.2)	0.583
Rural	189/427 (44.3)	50.7 (8.1; 144.8)		89/189 (47.1)	65/189 (34.4)	35/189 (18.5)	
*Season of blood withdrawal*							
Winter (Jan – Mar)	138/427 (32.3)	41.8 (8.1; 100.9)	<0.0001	84/138 (60.9)	38/138 (27.5)	16/138 (11.6)	<0.0001
Spring (Apr – Jun)	110/427 (25.8)	47.6 (12.1; 122.3)		63/110 (57.3)	36/110 (32.7)	11/110 (10.0)	
Summer (Jul – Sep)	62/427 (14.5)	63.7 (10.8; 174.3)		21/62 (33.9)	19/62 (30.6)	22/62 (35.5)	
Fall (Oct – Dec)	117/427 (27.4)	55.7 (12.0; 174.7)		45/117 (38.5)	45/117 (38.5)	28/117 (23.0)	
*Ethnicity*							
White	410/427 (96.0)	50.6 (10.0; 174.7)	<0.0001	198/410 (48.3)	137/410 (33.4)	75/410 (18.3)	0.005
Non-white	17/427 (4.0)	28.2 (8.1; 86.2)		15/17 (88.2)	1/17 (5.9)	1/17 (5.9)	
*Weight status*							
Normal	205/366 (56.0)	51.7 (13.5; 174.7)	0.032	95/205 (46.3)	60/205 (29.3)	50/205 (24.4)	0.011
Overweight	78/366 (21.3)	48.7 (10.0; 107.9)		42/78 (53.8)	28/78 (35.9)	8/78 (10.3)	
Obese	83/366 (22.7)	46.0 (10.8; 94.6)		48/83 (57.8)	27/83 (32.5)	8/83 (9.6)	
*Sun exposure*							
Low	50/277 (18.1)	34.6 (10.0; 97.7)	<0.0001	39/50 (78.0)	9/50 (18.0)	2/50 (4.0)	<0.0001
Moderate	77/277 (27.8)	52.4 (13.5; 174.7)		34/77 (44.2)	29/77 (37.7)	14/77 (18.1)	
Good	150/277 (54.1)	54.3 (18.1; 144.8)		63/150 (42.0)	47/150 (31.3)	40/150 (26.7)	
*Use of sunscreens*							
Regular	92/277 (33.2)	45.7 (10.0; 97.7)	<0.0001	58/92 (63.0)	29/92 (31.5)	5/92 (5.5)	<0.0001
Non regular	185/277 (66.8)	54.6 (18.1; 174.7)		78/185 (42.2)	56/185 (30.3)	51/185 (27.5)	
*Outdoor physical exercise*							
< 3 hours/weeks	173/277 (62.5)	47.7 (12.0; 174.3)	<0.0001	96/173 (55.5)	52/173 (30.1)	25/173 (14.4)	0.001
≥ 3 hours/weeks	104/277 (37.5)	59.5 (10.0; 174.7)		38/104 (36.5)	32/104 (30.8)	34/104 (32.7)	
*History of fractures*							
Positive	62/277 (22.4)	50.1 (13.5; 95.6)	0.376	30/62 (48.4)	23/62 (37.1)	9/62 (14.5)	0.231
Negative	215/277 (77.6)	50.2 (10.0; 174.7)		104/205 (48.4)	61/215 (28.4)	50/215 (23.2)	

The prevalence of severe vitamin D deficiency significantly differed in relation to season of blood withdrawal (winter 18/138 - 13.0%, spring 17/110 - 14.5%, summer 1/62 - 1.6%, fall 2/117 - 1.7%, p = 0.0001), ethnicity (white 32/410 - 7.8%, non-white 6/17 - 35.3%, p = 0.002), sun exposure (low 9/50 - 18.0%, moderate 7/77 - 9.1%, good 5/150 - 3.3%, p = 0.003), use of sunscreens (regular 13/92 - 14.1%, non regular 8/185 - 4.3%, p = 0.007) and outdoor physical exercise (< 3 hours/weeks 19/173 - 11.0%, ≥ 3 hours/week 2/104 - 1.9%, p = 0.006). The prevalence of severe vitamin D deficiency did not differ in relation to gender, residence, BMI, vitamin D and calcium intake and history of fractures (data not shown).

Table 3 shows the logistic regression analysis for the presumed predictors of vitamin D status. Blood sample obtained in winter-spring period, low sun exposure and regular use of sunscreens were significantly associated with an increased odds ratio (OR) of both vitamin D deficiency and hypovitaminosis D. Overweight-obese adolescents had a significantly increased OR of only hypovitaminosis D.

**Table 3:** Logistic regression for presumed risk factors for vitamin D deficiency (25-OH-D < 50 nmol/L) and hypovitaminosis D (25-OH-D < 75 nmol/L)

	**Vitamin D deficiency**				**Hypovitaminosis D**			
	**B (SE)**	**OR**	**C.I. 95%**	**p**	**B (SE)**	**OR**	**C.I. 95%**	**p**
*Gender*	-0.52 (0.28)	0.60	0.34-1.03	0.063	-0.43 (0.37)	0.65	0.31-1.36	0.251
(female vs male)								
*Residence*	0.01 (0.28)	1.01	0.58-1.76	0.975	0.27 (0.37)	1.30	0.63-2.70	0.477
(urban vs rural)								
*Season of blood withdrawal*	1.19 (0.30)	3.31	1.82-6.01	<0.0001	1.73 (0.40)	5.64	2.59-12.27	<0.0001
(winter-spring vs summer-fall)								
*BMI*	0.41 (0.29)	1.50	0.84-2.67	0.167	1.36 (0.48)	3.89	1.54-9.88	0.004
(normal vs overweight-obese)								
*Sun exposure*	1.57 (0.41)	4.78	2.15-10.63	<0.0001	1.78 (0.79)	5.94	1.25-28.17	0.025
(low vs moderate-good)								
*Use of sunscreens*	0.63 (0.31)	1.87	1.02-3.41	0.042	1.77 (0.54)	5.89	2.05-16.94	0.001
(regular vs non regular)								
*Outdoor physical exercise*	0.34 (0.30)	1.40	0.78-2.52	0.261	0.25 (0.38)	1.28	0.60-2.71	0.522
(< 3 vs ≥ 3 hours/week)								
*History of fractures*	-0.25 (0.33)	0.78	0.41-1.49	0.448	0.38 (0.49)	1.46	0.56-3.81	0.445
(positive vs negative)								
	*χ*^2^ = 49.62, p < 0.0001; Cox R^2^ = 0.173; Nagelkerke R^2^ = 0.231;				*χ*^2^ = 62.33, p < 0.0001; Cox R^2^ = 0.212; Nagelkerke R^2^ = 0.341;			
	Hosmer and Lemeshow test p = 0.482				Hosmer and Lemeshow test p = 0.564			

C.I.: Coefficient Interval.

**Figure 1:**
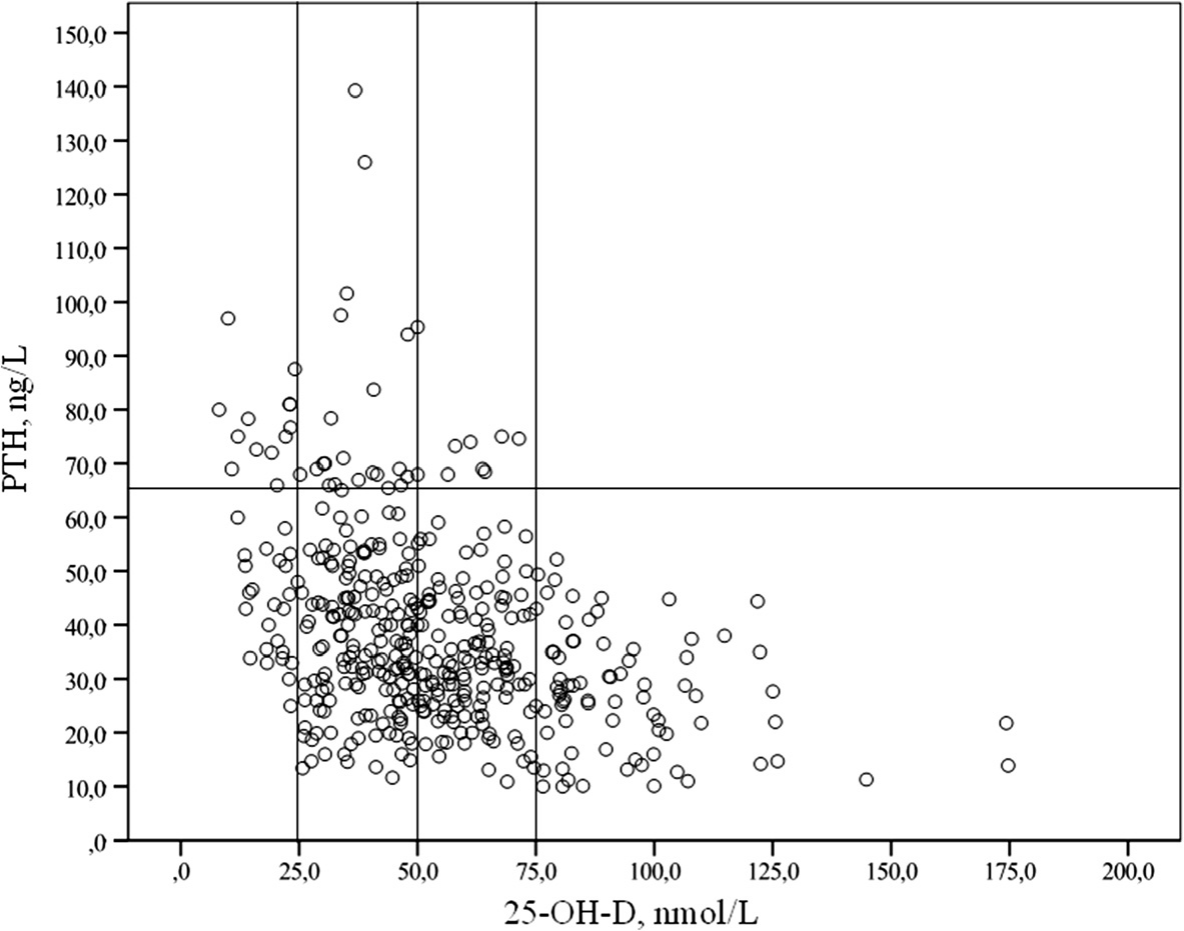
**Individual PTH levels according to serum 25-OH-D values.** Circles over the horizontal line (65.0 ng/L) are subjects with secondary hyperparathyroidism. Vertical lines represent the 25-OH-D cut-off of 25, 50 and 75 nmol/L.

**Figure 2:**
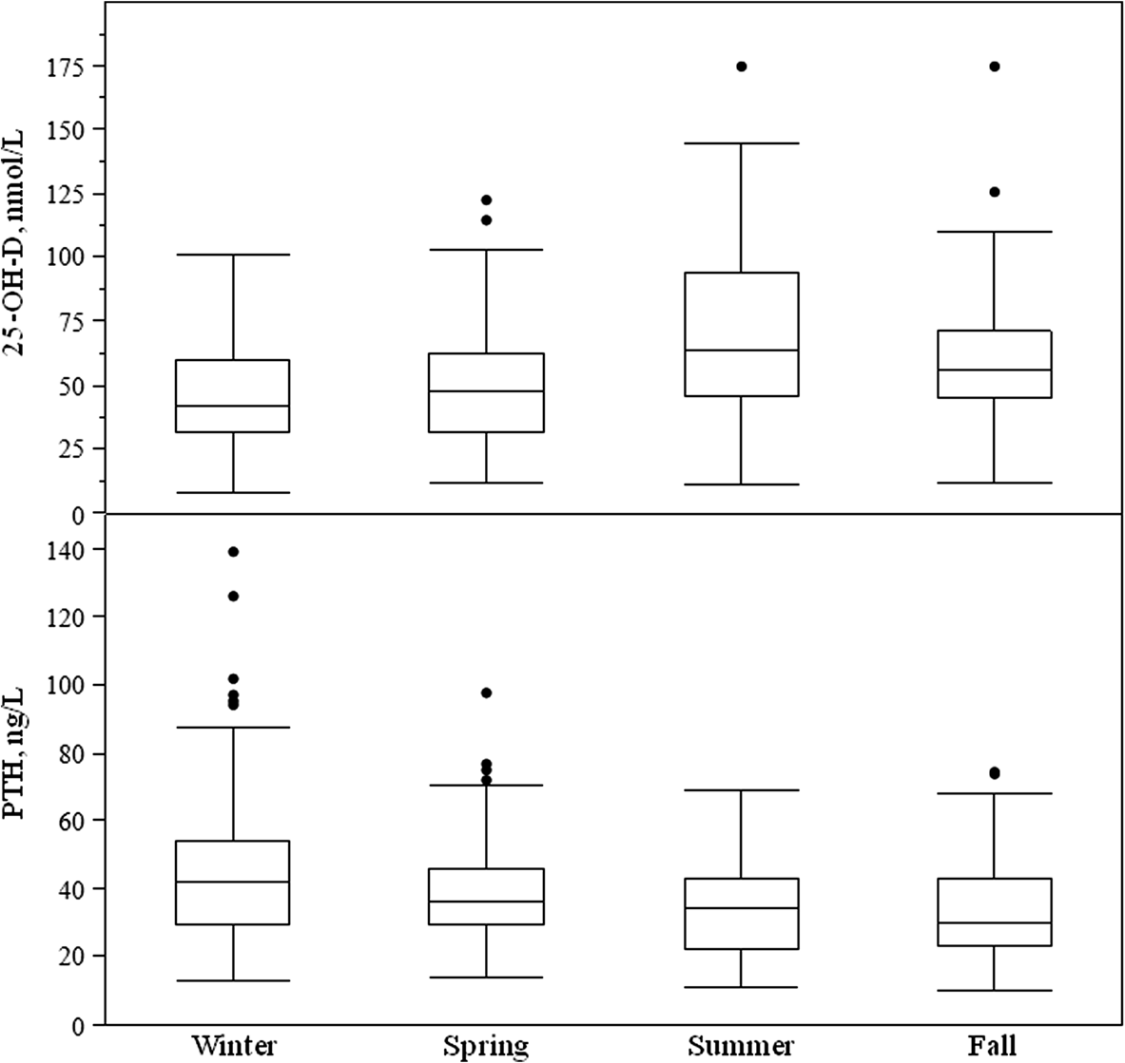
**Box plot of serum 25-OH-D and PTH levels according to the season of blood withdrawal.** p < 0.0001 between groups for both.

Non-white adolescents were at higher risk of severe vitamin D deficiency (OR 6.44, C.I. 95%: 2.24-18.56, p = 0.002) and vitamin D deficiency (OR 8.03, C.I. 95%: 1.81-35.56, p = 0.001) than white subjects, as assessed by Fisher’s exact test.Overall 10.3% (44/427) of the subjects had secondary hyperparathyroidism. In relation to vitamin D status, elevated PTH was detected in 16.4% (35/213) and 6.5% (9/138) of adolescents with deficiency and insufficiency, respectively. Notably, hyperparathyroidism was found in 34.2% (13/38) of subjects with severe vitamin D deficiency, while no subjects with vitamin D sufficiency had elevated PTH (Figure 1). Serum median PTH levels were significantly different depending on vitamin D status (deficiency 42.0 ng/L, range 11.7-139.3; insufficiency 33.0 ng/L, range 10.9-95.4; sufficiency 26.8 ng/L, range 10.0-52.2, p < 0.0001).25-OH-D and PTH levels significantly differed depending on season of blood withdrawal, as showed in Figure 2. 18.8% (26/138), 7.3% (8/110), 6.5% (4/62) and 5.1% (6/117) of adolescents showed secondary hyperparathyroidism during winter, spring, summer and fall, respectively (p = 0.001). Overweight-obese adolescents also showed increased prevalence of hyperparathyroidism in comparison with normal weight ones (22/161 - 13.7% vs 15/205 - 7.3%, p = 0.046 by Fisher’s exact test). Finally, non-white individuals had increased prevalence of hyperparathyroidism than white ones (6/17 - 35.3% vs 38/410 - 9.3%, p = 0.001 by Fisher’s exact test). The prevalence of hyperparathyroidism did not differ in relation to gender, residence, sun exposure, use of sunscreens, outdoor physical exercise and history of fractures (data not shown).

Blood evaluation during the winter-spring period was associated with an increased OR of hyperparathyroidism of 2.69 (C.I. 95% 1.29-5.59, p = 0.006 by Fisher’s exact test) in comparison with blood samples performed during summer-fall months. Non-white subjects had a significantly increased OR of hyperparathyroidism of 5.34 (C.I. 1.87-15.25, p = 0.004 by Fisher’s exact test).

1,25(OH)_2_D levels did not differ according to vitamin D status (n = 164 with vitamin D deficiency, median 1,25(OH)_2_D 101 pmol/L, range 11-273; n = 91 with vitamin D insufficiency, median 1,25(OH)_2_D 107 pmol/L, range 34-304; n = 49 with vitamin D sufficiency, median 1,25(OH)_2_D 94 pmol/L, range 49-299; p = 0.670).

Serum 25-OH-D levels were inversely related to PTH (r = -0.387, p < 0.0001) and BMI-SDS (r = -0.141, p = 0.007) but were not related to age, 1,25(OH)_2_D levels, calcium and vitamin D intake (data not shown). PTH levels did not correlate with calcium intake (r = 0.093, p = 0.124).

## Discussion

A small number of reports on vitamin D status of the Italian pediatric population have been published to date and data on adolescents are even more limited. Our study examined a large number of healthy adolescents, becoming to our knowledge the largest series focusing on this age group in Italy. The other two main studies dealing with vitamin D status and adolescents in Italy are by Chinellato et al. (n = 59, deficiency: 42.4%, insufficiency: 45.8%) [18] and Lippi et al. (n = 270, deficiency: 19.3%, insufficiency: 36.3%) [19]. Our results are in accordance with the first one, despite the small number of subjects enrolled in that series, while the study by Lippi et al. reports a remarkably lower prevalence of vitamin D deficiency and insufficiency. Notably, none of the two series accounted for supplementation and season of blood withdrawal, making it difficult to compare their results with ours.

Our results show a high prevalence of hypovitaminosis D among healthy adolescents, similarly to reports from other countries around the world [2,20-23].

Our data shows that the season of blood withdrawal was a significant predictor of vitamin D status, with winter and spring being the seasons associated with lower median 25-OH-D levels, in the range of deficiency. This is in accordance with other reports [20,21,24]. At our latitude, sun exposure is effective in promoting vitamin D activation from May through September only [13]. However, the high prevalence of hypovitaminosis D we found during winter and spring suggests that the amount of vitamin D produced and stored from May to September is probably not sufficient to guarantee an optimal vitamin D status in unsupplemented healthy adolescents during the remaining months of the year, particularly in presence of factors limiting summer sun exposure. Indeed, adolescents undergoing blood withdrawal during winter and spring were at a three times higher risk of vitamin D deficiency and at a five times higher risk of hypovitaminosis D compared to those evaluated during summer or fall. Moreover, adolescents with low summer sun exposure were at a higher risk of vitamin D deficiency and hypovitaminosis D (OR 4.78 and 5.94, respectively). This result may reflect the growing attitude of Italian adolescents to spend a considerable amount of time on indoor activities, as recently reported by the Italian Society of Pediatrics [25].

The use of sunscreens resulted another predictor of vitamin D status. Regular use of sunscreen was associated to an increased OR of 1.87 and 5.89 of vitamin D deficiency and hypovitaminosis D, respectively. These results may be explained by the fact that 90% of vitamin D levels depends on cutaneous synthesis and that the use of a sunscreen with an SPF of 15 decreases vitamin D synthesis in the skin by 99% [14]. Finally, also outdoor physical exercise was related to vitamin D status. Adolescents performing < 3 hours/week of outdoor activities had higher prevalence of vitamin D deficiency and insufficiency, but a non significant increase in the risk of hypovitaminosis D. However, we did not account for the seasonal distribution of outdoor physical exercise, possibly explaining these results. Other studies suggest that physical exercise is a determinant of vitamin D status and it is well known that adolescents do not practice physical activity regularly [2,20,24].

Considering the aforementioned already scarce attitude to sun exposure in adolescence, these results stress the importance of sensible sun exposure. While there is no agreement on the amount of time that adolescents can safely spend in sunlight without sun protection [26], pediatricians should encourage regular sun exposure of legs and arms at least 2 times/week for at least 15 minutes without the use of sunscreens. This is considered to be a sufficient amount of time to promote adequate cutaneous vitamin D synthesis [27,28].

We found that non-white adolescents had an eight times higher risk of vitamin D deficiency and a six times higher risk of severe deficiency, compared to white subjects. Similar results have been reported by other studies [20,29]. Thus, non-white adolescents are a high-risk group for hypovitaminosis D and clinicians should tackle them with vitamin D supplementation, particularly during winter.

Finally, BMI was also shown to significantly influence vitamin D status, with overweight and obese adolescents being at a higher risk of vitamin D deficiency (OR 1.50) and hypovitaminosis D (OR 3.89). These results are in accordance with previous studies [20,24,29]. Obesity-associated hypovitaminosis D is likely due to the decreased bioavailability of vitamin D because of its deposition in body fat compartments [30]. Moreover, adiponectin has been recently identified as a key plasma protein that links vitamin D deficiency to pediatric obesity [31]. Pediatricians should therefore encourage adolescents to adopt a healthier lifestyle as a whole, promoting weight loss, physical exercise and outdoor activities. Vitamin D supplementation should be considered particularly in the presence of poor compliance with lifestyle modification.

Other factors investigated such as gender, place of residence and vitamin D and calcium dietary intake did not influence vitamin D status. In particular, the role of gender in the determination of vitamin D status is not clear. Other studies may have been influenced, at least in part, by cultural attitudes towards sun exposure and concealing clothes [32,33]. In general, our results are in line with other findings [24,34,35]. With reference to residence, our results are not in accordance with those of other studies such as that of Saintonge et al. [29]. However, the dense concentration of buildings impairing sunlight exposure in metropolitan areas may not be found in Tuscany. Regarding dietary intake of vitamin D, Italian typical diet does not include vitamin D-rich foods such as fish (salmon, sardines, tuna, mackerel) or shiitake mushrooms, nor fortified foods. Particularly, in Italy milk is not routinely fortified with vitamin D. This may explain the different results obtained in other countries [23,36].

Regarding our results on the relationship between 25-OH-D and PTH, we found a remarkably high prevalence of hyperparathyroidism in our series of otherwise healthy adolescents. Adolescence is a crucial period for the achievement of PBM, therefore hyperparathyroidism could affect bone health in the short and longer term [37]. As expected, adolescents with 25-OH-D levels in the range of severe deficiency were at a higher risk of hyperparathyroidism. Interestingly, we found hyperparathyroidism in 6.5% of adolescents with vitamin D insufficiency, while we did not report any case among adolescents with vitamin D sufficiency. Even though the majority of the pediatric societies recommend 50 nmol/L as the cut-off for sufficiency and despite the ongoing debate in literature [38], these results support our choice to use the cut-off proposed by the Endocrine Society. Other Authors obtained similar results in studies based on pediatric population [39] and there is an increasing tendency to apply these cut-off in the pediatric age group [8].

In our series PTH levels were significantly related to seasons, with a winter-time prevalence of secondary hyperparathyroidism three times higher than the prevalence during summer. A comparable result was obtained by other Authors [24,40] and reflects the seasonal variation of 25-OH-D levels. Weight status and ethnicity significantly influenced PTH levels in addition to 25-OH-D concentrations, confirming the importance of promoting an adequate vitamin D status in high-risk group such non-white and overweight-obese adolescents.

Finally, we confirmed that 1,25(OH)_2_D evaluation is not useful to diagnose hypovitaminosis D because 1,25(OH)_2_D levels did not differ according to vitamin D status [8].

Some strong points of our cross-sectional study were the conspicuous number of healthy adolescents enrolled, that makes this the largest Italian study on this age group. Furthermore, all subjects were not receiving vitamin D supplementation, allowing us to consider a large number of different vitamin D status predictors. The main limitation of our study was the lacking of longitudinal evaluation of vitamin D status that could clarify the role of the proposed risk factors of hypovitaminosis D in relation to seasonality. Some recall bias possibly occurring during data collection through questionnaire and the indirect assessment of sun exposure and physical activity may represent other limitations. Auxological evaluation and questionnaire were not performed in all adolescents. Finally, the subjects enrolled were not a random sample and therefore our series may not represent the whole Italian population of adolescent age.

## Conclusions

Italian adolescents are at significant risk of vitamin D deficiency and insufficiency. Winter-spring period, low sun exposure, regular use of sunscreen, < 3 hours/week outdoor physical exercise, weight excess and non-white ethnicity significantly affected vitamin D status. Moreover, adolescents with hypovitaminosis D may develop secondary hyperparathyroidism that may affect bone mass accrual and the acquisition of PBM. In the light of these results, pediatricians should tackle risk factors of hypovitaminosis D, favoring a healthier lifestyle in the adolescence age group. Vitamin D supplementation should be considered in groups at higher risk.

## Abbreviations

1,25(OH)_2_D: 1,25-dihydroxyvitamin D; 25-OH-D: 25-hydroxyvitamin D; BMI: Body mass index; OR: Odds ratio; PBM: Peak bone mass; PTH: Parathyroid hormone; SPF: Sun protector factor; 25-hydroxyvitamin D: nmol/L = ng/mL * 2.496; 1,25-dihydroxyvitamin D: pmol/L = pg/mL * 2.6; Parathyroid hormone: pg/mL = ng/L.

## Competing interests

The authors declare that they have no competing interests.

## Authors’ contributions

FV and MDP designed the study, acquiring and analyzing data. FV, MDP and MF drafted the manuscript. PE carried out the biochemical assays. FV performed statistical analysis. GS revised the manuscript. All authors read and approved the final manuscript.

## Authors’ information

FV: Pediatric Unit, Campo di Marte Hospital, Lucca, Italy.

MDP, MF, GS: Pediatric Clinic, University-Hospital Pisa, Italy.

PE: Nuclear Medicine Unit, University-Hospital Pisa, Italy.
